# Effect of EPA on Hsp90 and GRα protein expression in multiple myeloma drug-resistant cells

**DOI:** 10.1186/s12885-021-08804-6

**Published:** 2021-10-03

**Authors:** Shenghao Wu, Yuemiao Chen, Xueshuang Wang, Shanshan Weng, Wenjin Zhou, Zhen Liu

**Affiliations:** Department of Hematology, The Second Affiliated Hospital of Shanghai University (The Dingli Clinical Institute of Wenzhou Medical University, Wenzhou Central Hospital), No.252 East Baili Road, Lucheng District, Wenzhou, 325000 Zhejiang Province China

**Keywords:** Myeloma, EPA, Heat shock protein-90, GRα, Apoptosis

## Abstract

**Background:**

Approximately 20% of MM patients harbor glucocorticoid (GC) resistance and are not responsive to therapeutic effect. Chaperoneheat-shock proteins Hsp90 is needed for ligand docking, The imbalance of Hsp90/GRα (glucocorticoid receptor α) may be an important cause of GC resistance. Recent studies have indicated that EPA could repress cancer cell growth by regulating critical influential factors in progression of cancer, consisting of resistance to drugs, chemosensitivity. The aim of the present study was to test the cytotoxic effects of EPA alone or EPA + Dexamethasone in dexamethasone-resistant MM cell (MM.1R) and investigate whether DHA can induce apoptosis and reverse acquired glucocorticoid resistance in dexamethasone-resistant MM cell (MM.1R).

**Methods:**

Cell Counting Kit-8 (CCK-8) was used to detect the proliferation of MM.1R cells after treating with EPA alone and EPA combined with DEX. Mitochondrial membrane potential was measured by flow cytometry and GRα and Hsp90 protein expression were assessed by western blot analysis.

**Results:**

EPA alone was able to inhibit cell proliferation as evidenced by CCK-8 assay and the tumor growth was remarkably suppressed by EPA + Dexamethasone, Cell apoptosis after EPA treatment was obviously observed by Flow cytometry analysis of the mitochondrial membrane potential. Analysis of Hsp90 and GRα proteins in MM.1R cells incubated with EPA revealed down-regulation of Hsp90 and up-regulation of GRα. Accordingly, the Hsp90/GRα ratio was significantly decreased with the increase of EPA concentration.

**Conclusions:**

EPA might be used as a new effective treatment for reversal of glucocorticoid-resistance in multiple myeloma.

## Introduction

Multiple myeloma (MM) is a hematological tumor of terminally differentiated bone marrow (BM)-resident B lymphocytes referred to as plasma cells (PCs) [1]. Despite improvementsin the understanding of its biology, MM is still an incurable disease due to treatment-related mutations and drug resistance [2]. Therefore, new treatment strategiesthat target either metastatic plasma cells or the microenvironment of bone marrow can be considered for MM. Glucocorticoids (GCs) are an important agentemployed in all therapeutic formulations for treating leukemia, as well as multiple myeloma. The killing mechanisms of glucocorticoids include triggering the apoptosis of myeloma cells and decreasing poly (ADP ribose) polymerase cleavage, mitochondrial transmembrane potential and caspase 3 expression [3]. Nevertheless, approximately 20% of patients harbor glucocorticoid resistance and are not responsive to treatment. Moreover, those who are responsive may develop glucocorticoid resistance along the treatment time course, resulting in relapse and a very dismal prognosis. However, some new findings provide new tools for overcoming glucocorticoid resistance [4]. Enhancing glucocorticoid sensitivity is a growing challenge in the treatment of MM. Diverse mechanisms of resistance to glucocorticoids have been reported in MM. Functional abnormalities of the glucocorticoid receptor underlie the occurrence of glucocorticoid resistance. A truncated glucocorticoid receptor (GR) without the C-terminal hormone docking domain has been reported in dexamethasone (DEX)-resistant MM cells [5]. There are two primary GR isoforms (*α*and *β*), with the GR*α* isoform being the only GC functional receptor [6]. Studies have suggested that GR*β* has a prominent negative influence on GR*α* via GR*α*/GR*β* heterodimer formation, which prevents GR*α* action [7, 8]. Unstimulated GRs remain in the cytoplasm as heterocomplexes with chaperone heat-shock proteins, Hsp90, and Hsp70. Hsp90 is needed for ligand docking, and Hsp70 is believed to facilitate ligand delivery to Hsp90 [9]. However, the overexpression of Hsp90 has been documented to negatively modulate GR promoter activity, repress GR transcription, and decrease GR inclusion in cytoplasm complexes [10, 11].

Eicosapentaenoic acid (EPA) and docosahexaenoic acid (DHA) are long-chain PUFAs. These PUFAs play pivotal functions in the cell as structural compartments of cellular membranes and as mediators of intracellular metabolic cascades. PUFAs have advantageous activities in diverse chronic conditions, such as neurodegenerative disorders, inflammatory conditions, cardiovascular diseases, and cancers [12–15]. Studies have indicated that EPA, as well as DHA, repress cancer cell growth and survival by regulating critical influential factors in the progression of cancer, including drug resistance, chemosensitivity and angiogenesis [16–19]. The mechanism of cancer cell suppression by PUFAs involvesthe induction of apoptosis [20, 21]. Lipid peroxidation causes irreversible cell damage [22–24] and modulates gene expression, including that oftranscription factors [25–28].

A previous study reported Hsp90 overexpression in multiple malignant cell types, including MM. To our knowledge, no previous studies have examined whether DHA can decrease Hsp90 inclusion in the GR complex, induce apoptosis, and reverse acquired glucocorticoid resistance in dexamethasone-resistant MM cells (MM.1R). Considering the anticancer capacity of DHA along with its limited side effects on healthy cells, we explored the effect of DHA on Hsp90 and GR*α* expression in multiple myeloma cells.

## Materials and methods

### Reagents and antibodies

DEX-resistant human multiple myeloma cells (MM.1R) were purchased from the Cell Bank of the Chinese Academy of Sciences (Shanghai, China) and were preserved in our laboratory. Then, they were removed from liquid nitrogen and characterized. Conventional resuscitation and subculture of MM.1R cells were performed. RPMI 1640 medium and fetal bovine serum (FBS) were purchased from Gibco (NY, USA). EPA and DEX were purchased from Sigma, cell cycle detection kits and flow cytometry apoptosis detection kits were purchased from KeyGEN Biotech (Nanjing, China). anti-GR*α* antibodies (11000), anti-Hsp90 antibody and GAPDH antibody were purchased from Abcam, the protein lysate, BCA protein concentration determination kit, fluorescent secondary antibody, ECL chemiluminescence kit, and CCK-8 kit were purchased from BiYunTian Biotech Company (Shanghai, China).

### Cell culture experiments

MM.1R cells were inoculated in DMEM enriched with fetal bovine serum (10%), streptomycin (100 U/ml), and penicillin (100 U/ml) and then incubatedin an incubator programmed at 37 °C and 5% CO_2_. The growth media was refreshed every 2–3 days, and passaging of the cells was performed accordingly, logarithmic-phase cells were selected for downstream experiments.

### Group allocation

DEX was suspended in 0.05% DMSO, dissolved to form a stock solution at a concentration of 10 mmol/l, and then diluted 1000 times with RPMI 1640 medium. The final DEX concentration was adjusted to 10 μmol/L. EPA reserve solution (100 mmol/L) was prepared with anhydrous ethanol. The working solution (10, 20, 50, 100 μmol/L) was then preparedby diluting the stock solution with RPMI1640. In the combined dosing group, different concentrations of EPA were incubated for 12 h. An additional 10 μmol/L DEX was introduced, and incubation was continued for 24 h, and then further experiments were performed. Equal volumes of medium were introduced to the control group. The growth of the control cells was assessed in medium with a similar concentration of DMSO or anhydrous ethanol as the DHA-containing medium. EPA treatments at concentrations of 10–50 μmol/L remarkably suppressed cell proliferation.

### Cell proliferation evaluation by the CCK-8 assay

Cells in the logarithmic growth phase were collected. Digestion of the cells was performed using 0.25% trypsin, followed by cell suspension preparation. We adjusted the cell density to 1 × 10^5^ cells/ml. Thereafter, the cells were plated in 96-well plates (200 μl/well). The experiments were performed in 5 replicate wells. The cells were grown for 24 h in an incubator programmed at 37 °C, 5% CO2, and saturated humidity. After the cells were allowed to grow and adhere for 24 h, the drug solution was added to the cells at various concentrations. CCK-8 reagent (10 μl) was added to each well, the samples were thoroughly mixed, and the plates were placed in an incubator for 2 h. Absorbance values (OD values) were determined at 450 nm using an enzyme standard instrument and used in the following formula:cell growth inhibition rate = (test OD value − blank OD value)/(control OD value − blank OD value) × 100%. Bar graphs of the final value were generated, and the results were analyzed. The experiment was replicated three times, and the results were averaged.

### Flow cytometry analysis of the mitochondrial membrane potential

A mitochondrial membrane potential assay kit with JC-1 was employed to evaluate the mitochondrial membrane capacity. MM.1R cells in the logarithmic growth phase were plated (1 × 10^5^ cells/well) in 6-well plates (2 ml per well), and then different concentrations of drugs were added. Cells were collected through centrifugation 48 h after the treatment. Samples were then rinsed with ice-cold PBS. Staining of the cells with 500 μl JC-1 working solution was carried out at 37 °C for 15 min, and the cells were collected after sedimentation. After that, 500 μl preheated JC-1 assay buffer was used to resuspend the cells, which were analyzed on a flow cytometer. The assay was replicated three times, and the average result was used.

### Western blot analysis of GR*α* and Hsp90 protein expression

The cells were processed as described above. The MM.1R cells were treated as stated above. After treatment for 24 h, the cells were collected, and protein purification was conducted using RIPA lysis buffer. The culture supernatants was collected and centrifuged to remove any cell debris. Quantitation of the total protein was conducted using the BCA™ protein assay (Pierce). Fractionation of the total protein was performed using SDS-PAGE. After that, the proteins were transfer-embedded onto PVDF membranes. Blocking of the nonspecific sites was performed via a1-h incubation of the protein-embedded membranes with Tris-buffered saline-Tween 20 (TBST) enriched with 3% bovine serum albumin at room temperature. Thereafter, incubation with primary antibody was conducted overnight in an incubator set at 4 °C. Subsequently, the samples were rinsed three times with TBST, and then conjugation with the secondary antibodies at a 1:5000 dilution for 2 h was performed via incubation at room temperature. After washing, color development of the protein bands was carried out using ECL for 3–5 min. The band intensity for each protein was scanned on a scanner and analyzed using Image Quant 5.1 image processing software. GAPDH was employed as a reference gene. Relative quantitative analysis was also performed using Image Quant 5.1 software. The ratio of the optical density of the target protein to that of GAPDH was computed as the relative expression level of the target protein in the samples. This experiment was performed three times, and the results are presented as averaged values.

### Statistical analyses

SPSS 16.0 software was applied in statistical analyses. All data are indicated as the mean ± standard deviation (SD), and a two-sample independent t test was used. *P* < 0.05 signified statistical significance.

## Result

### The influence of EPA on the proliferation of MM.1R cells

EPA treatment at concentrations of 10–50 μM remarkably suppressed cell proliferation (*P* < 0 .05 for all). In comparison to the single-drug treatments, the combination of EPA and DEX remarkably repressed cell growth. There was a statistically significant difference between the two groups at the same time point (*P* < 0.05). At a given time point (24 or 48 h), different concentrations of EPA had significantly different effects on the proliferation inhibition rate of MM.1R cells (Table 1).

**Table 1:** The influence of eicosapentaenoic acid (EPA) combined with dexamethasone (DEX) on the growth of MM.1R cells (%,^−^ x ±s)

Group	The rate of cell proliferation inhibition	
	24 h	48 h
Control	0	0
10 μmol/L DEX	0	5.47 ± 0.25^ac^
10 μmol/L EPA	8.45 ± 0.57^a^	12.07 ± 0.94^ac^
20 μmol/L EPA	15.24 ± 1.27^a^	24.59 ± 1.29^ac^
50 μmol/L EPA	21.97 ± 2.14^a^	35.47 ± 2.416^ac^
50 μmol/L EPA + 10 μmol/L DEX	38.78 ± 2.73^ab^	68.24 ± 2.02^abc^

Note: Compared with the blank control group, ^a^*P* < 0.05; compared with the 50 μmol/L EPA group, ^b^*P* < 0.05; compared with the 24 h group, ^c^*P* < 0.05. *n* = 3

### The influence of EPA on the mitochondrial membrane potential of MM.1R cells

**Fig. 1:**
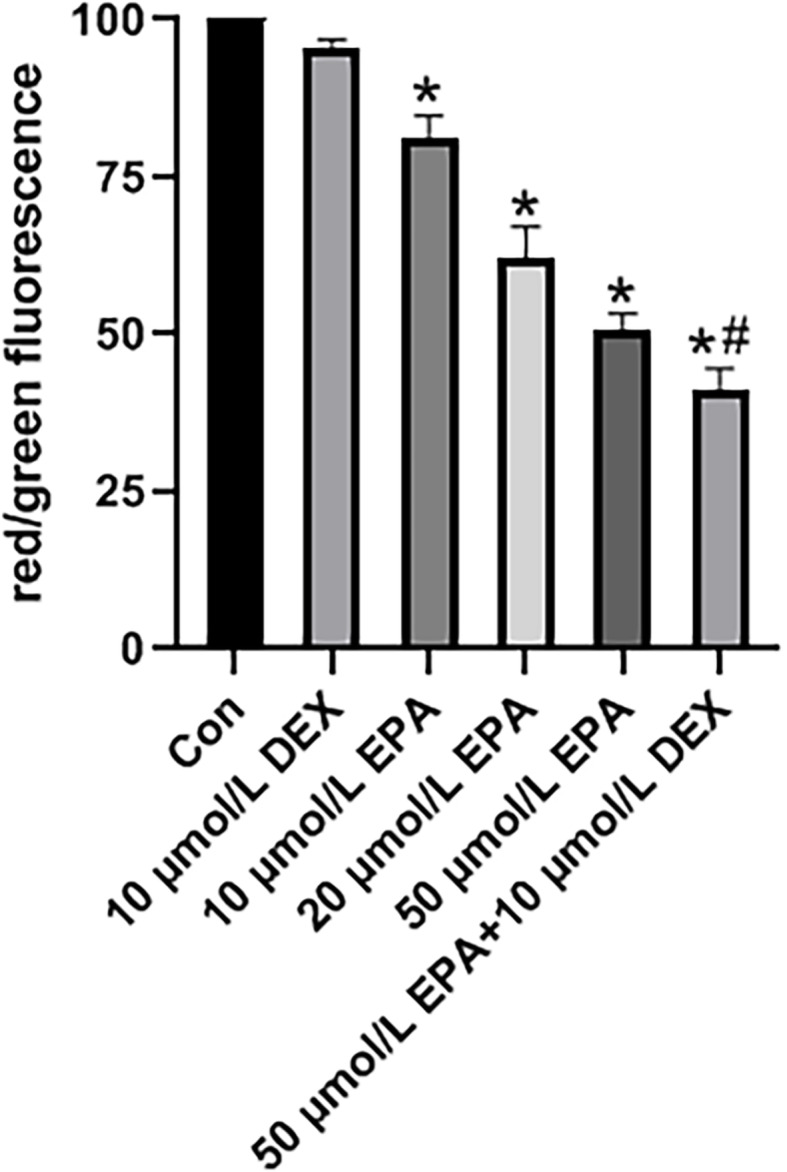
Effect of EPA on MM.1R apoptosis through the mitochondrial pathway. Cells were exposed to 10 μmol/L DEX, 10 μmol/L EPA, 20 μmol/L EPA, 50 μmol/L EPA, and 50 μmol/L EPA + 10 μmol/L DEX for 48 h. Mitochondrial membrane potential was measured via JC-1 staining. Illustrative images of JC-1-derived fluorescence in MM.1R cells. Quantitative evaluation of the ratio of red/green fluorescence.*P < 0.05 vs. control; #*P* < 0.05 vs. 50 μmol/L EPA, *n* = 3

One mechanism through which apoptosis occurs entails mitochondrial membrane integrity loss, as well as the loss of transmembrane potential (i.e., collapse of Δψm). Changes in mitochondrial membrane potential, ΔΨm, are a pivotal step in cells undergoing apoptosis. We explored the impact of EPA on mitochondrial membrane potential via astaining approach with the fluorescent membrane-permeant JC-1 dye and subsequent flow cytometric analysis. JC-1 accumulation is shown by red fluorescence, the JC-1 monomer is indicated by green fluorescence, and the merged image combines the red and green images. Confocal microscopy demonstrated that EPA reduced red JC-1 fluorescence and increased green JC-1 fluorescence, leading to a reduction in the red/green fluorescence ratio. The fluorescence intensity gradually decreased with the progressive increase of EPA concentration and mitochondrial membrane potential in MM.1R cells drop to the maximum extent in the combined treatment group of EPA + dexamethasone(*P* < 0.05) (Fig. 1).

### Western blot verification of Hsp90 and GR*α* protein expression

**Fig. 2:**
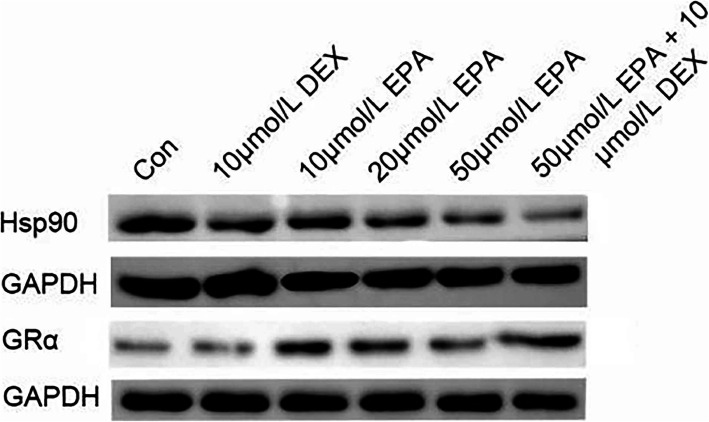
Western blot analysis of Hsp90 and GRα protein expression. MM.1R cells were treated with different concentrations of EPA for 24 h, and Western blot analyses were performed with antibodies against Hsp90 and GRα, or GAPDH. Results were representative of 3 independent experiments with similar results

MM.1R cells were simultaneously treated with different concentrations of EPA for 24 h. The Hsp90 content decreased gradually with increasing EPA concentration, and the expression of Hsp90 decreased significantly in the combined EPA + dexamethasone group. GRα increased gradually with increasing EPA concentration, and when EPA was combined with dexamethasone. The effect appeared to be dose dependent. Accordingly, the ratio of Hsp90 to GRα expression decreased gradually with increasing EPA concentration (*P* < 0.05) (Table 2, Fig. 2).

**Table 2:** The influence of EPA on Hsp90 and GRα protein expression (%, ^−^ x ±s)

Group	Hsp90	GR*α*	Hsp90/GR*α*
Control	2.91 ± 0.03	0.85 ± 0.03	3.39 ± 0.11
10 μmol/L DEX	2.90 ± 0.04	0.87 ± 0.02	3.32 ± 0.12
10 μmol/L EPA	2.53 ± 0.05^a^	1.76 ± 0.03^a^	1.44 ± 0.05^a^
20 μmol/L EPA	2.05 ± 0.03^a^	2.31 ± 0.04^a^	0.88 ± 0.02^a^
50 μmol/L EPA	1.83 ± 0.05^a^	2.76 ± 0.04^a^	0.66 ± 0.02^a^
50 μmol/L EPA + 10 μmol/L DEX	1.58 ± 0.03^ab^	2.97 ± 0.08^ab^	0.53 ± 0.01^ab^

Note: Compared with the blank control group, ^a^*P* < 0.05; compared with the 50 μmol/L EPA group, ^b^*P* < 0.05. n = 3

## Discussion

Herein, the efficacy of EPA in triggering cell apoptosis was explored in MM cells. We established that treatment of MM cells using different concentrations of EPA causes remarkable apoptotic death in time-dependentand dose-dependent manners. EPA reduced the mitochondrial membrane potential in MM cells, implying that the apoptosis of MM cells was partly caspase-dependent. The proliferation assay showed that EPA exhibited a comparable inhibitory role with DEX in MM.1R cells. The differences in metabolism and structure between malignant cells and the corresponding healthy cells account for the reported effect of EPA on malignant plasma cells, making malignant cells more vulnerable to the cytotoxic influence of EPA. EPA destroys mitochondria to reduce mitochondrial membrane potential and ultimately induces apoptosis in MM.1R cells. Intriguingly, the effects of EPA on cell apoptosis in acute myeloid leukemia cells were linked to mitochondrial metabolism disruption, the stimulation of mitochondrial swelling, and the reduction of mitochondrial membrane potential and were correlated with elevated oxidative stress and the dysregulation of Nrf2 [29].

Chaperone Hsp90 has been documented as a pivotal factor participating in GR complex stabilization, and only after dislocation of Hsp90 is GR translocated to the nucleus [30]. The imbalance of Hsp90/GRα may be an important cause of GC resistance, an elevated Hsp90/GRα ratio affects the sensitivity to ligands at a step that occurs after receptor initiation [31]. Hence, we assessed Hsp90 linked to GRα in MM.1R. Hsp90 expression decreased gradually with increasing EPA concentration. We additionally assessed the ratio of Hsp90 to GRα expression levels and established that the ratio decreased gradually with increasing EPA concentration. These data suggest that EPA decreases Hsp90 protein expression in the GR complex and influences GR function, leading to its enhanced responsivity to glucocorticoids.

In summary, our observations suggest that EPA has potential applications as a new effective treatment for the reversal of glucocorticoid resistance in multiple myeloma. The fact that EPA is active at physiological concentrations could necessitate further investigations of its clinical efficacy in MM patients.

## Data Availability

The datasets used and/or analysed during the current study available from the corresponding author on reasonable request.
